# Period of Boar Ejaculate Collection Contributes to the Yearly Intra-Male Variability of Seminal Plasma Cytokines

**DOI:** 10.3390/biology9050105

**Published:** 2020-05-20

**Authors:** Lorena Padilla, Xiomara Lucas, Inmaculada Parrilla, Cristina Perez-Patiño, Heriberto Rodriguez-Martinez, Jordi Roca, Isabel Barranco

**Affiliations:** 1 Department of Medicine and Animal Surgery, Veterinary Science, University of Murcia, 30100 Murcia, Spain; lorenaconcepcion.padilla@um.es (L.P.); xiolucas@um.es (X.L.); parrilla@um.es (I.P.); cristina.perez6@um.es (C.P.-P.); isabel.barranco@udg.edu (I.B.); 2 IMIB-Arrixaca, Regional Campus of International Excellence, Campus Mare Nostrum, University of Murcia, 30100 Murcia, Spain; 3 Department of Biomedical and Clinical Sciences (BKV), Linköping University, SE-58185 Linköping, Sweden; heriberto.rodriguez-martinez@liu.se; 4 Department of Biology, Faculty of Sciences, University of Girona, 17003 Girona, Spain

**Keywords:** cytokines, intra-male variability, pig, seasonality, seminal plasma

## Abstract

The concentrations of cytokines in seminal plasma (SP) fluctuate over time in healthy males, weakening their practical usefulness as diagnostic tools. This study evaluated the relevance of intra-male variability in SP cytokines and to what extent the period of the year when ejaculate is collected contributes to such variability. Thirteen cytokines (GM-CSF, IFNγ, IL-1α, IL-1β, IL-1ra, IL-2, IL-4, IL-6, IL-8, IL-10, IL-12, IL-18, and TNFα) were measured using the Luminex xMAP^®^ technology for 180 SP samples of ejaculate collected over a year from nine healthy and fertile boars. The SP samples were grouped into two annual periods according to decreasing or increasing daylight and ambient temperature. Intra-male variability was higher than inter-male variability for all cytokines. All SP cytokines showed concentration differences between the two periods of the year, showing the highest concentration during the increasing daylength/temperature period, irrespective of the male. Similarly, some cytokines showed differences between daylength/temperature periods when focusing on their total amount in the ejaculate. No strong relationship (explaining more than 50% of the total variance) was found between annual fluctuations in SP-cytokine levels and semen parameters. In conclusion, the period of the year during which ejaculates were collected helps explain the intra-male variability of SP-cytokine levels in breeding boars.

## 1. Introduction

Seminal plasma (SP), a complex fluid mainly secreted from the epididymis and the accessory sex glands, contains many bioactive components, particularly proteins and peptides [1,2,3]. These SP proteins and peptides play a key role in regulating both sperm function, i.e., fertilizing ability, and even the uterine immune environment once deposited into the female genital tract [4,5,6]. Consequently, the SP proteome is currently being explored for potential biomarkers of male (in)fertility [7,8,9].

The SP proteome includes cytokines, small proteins that have acquired growing reproductive interest in recent years because some of them are directly involved in the regulation of the immune environment of the female genital tract and, thereby, they play a key role in development and embryo implantation [10,11]. Moreover, some SP cytokines are also involved in sperm function [12,13], including fertilizing ability [14]. These findings open the possibility for some SP cytokines to be potential biomarkers of male fertility performance. Therefore, the measurement of SP-cytokine concentrations is being increasingly used to identify males with sub- or infertility [15,16,17]. However, the SP-cytokine levels vary among healthy males, as demonstrated in humans [18] and pigs [19], albeit the factors that explain this individual variability are yet unknown [18], thus blurring their practical usefulness as a diagnostic tool. Recently, studies in humans indicated that annual fluctuations in environmental conditions could influence the circulating blood levels of cytokines in healthy [20] and sick [21] men. In this context, the present study tested the hypothesis that year seasonal variations of increasing or decreasing ambient daylength and air temperature, during which ejaculates were collected from breeding boars, influences SP-cytokine concentrations. Being derived from wild boar, the domesticated pig is a species generally considered reproductively non-seasonal but still showing clear seasonal breeding/fertility, including clear differences in semen variables, even in breeding male pigs housed in buildings with a controlled environment [22,23,24,25]. To accomplish this goal, the concentrations of 13 cytokines were measured in 180 SP samples harvested from ejaculates collected over one year from nine healthy and fertile boars used in artificial insemination (AI) programs.

## 2. Materials and Methods

### 2.1. Animals, Ejaculates, and Seminal Plasma Collection

All procedures involving animals were performed according to international guidelines (Directive 2010/63/EU), and were approved in advance by the Bioethics Committee of Murcia University (research code: 639/2012).

Ejaculate donors were healthy, sexually mature Pietrain breeding boars aged between 18 and 22 months at the beginning of the experiment. The boars were housed in individual pens in an AI center belonging to Topics-Norsvin España and located in Calasparra, Murcia, Spain (38°15′ N, 1°35′ W). The environmental conditions of this locality during the experimental timeframe were characterized by daylight varying from 9 h 30 min at the winter solstice to 14 h 50 min at the summer solstice, and the maximum and minimum temperatures averaged 32.5 °C in July (summer) and 3.6 °C in January (winter), respectively. The building where boars were housed was equipped with evaporative coolers to maintain air temperature within comfort zones and an artificial lighting regime of 16 h of light per day. The boars were provided with water ad libitum and fed with commercial feedstuff specially formulated to fulfill the nutritional requirements of AI boars. The boars followed regular semen collections (an ejaculate every 3–4 d) to prepare liquid semen AI doses. Volume (measured directly in the graduated collection vessel), sperm concentration (counted by photometry), sperm morphology, and sperm subjective motility (assessed using a phase-contrast microscope) of each ejaculate were recorded in the AI center. All ejaculates used in the experiment fulfilled standards of quantity and sperm quality thresholds for liquid semen AI doses, namely, more than 100 mL of volume, more than 200 × 10^6^ spermatozoa per mL, more than 70% showing progressive motility, and more than 75% displaying normal morphology. Seminal plasma was harvested immediately after ejaculate collection by double centrifugation (1500× *g*, for 10 min at room temperature (RT); Rotofix 32A; Hettich Zentrifugen, Tuttlingen, Germany). The second supernatant was microscopically confirmed to be cell-free.

### 2.2. Cytokine Measurement

The concentrations of cytokines in SP samples were measured using the Luminex xMAP^®^ technology and a porcine cytokine/chemokine pre-coated magnetic bead panel (Cat#PCYTMG-23K-13PX Merck Millipore, Burlington, MA, USA). This panel allows the simultaneous measurement of 13 cytokines, namely, granulocyte-macrophage colony-stimulating factor (GM-CSF), interferon-gamma (IFNγ), interleukin (IL)-1α, IL-1β, IL-1ra, IL-2, IL-4, IL-6, IL-8, IL-10, IL-12, IL-18, and tumor necrosis factor-α (TNFα). The protocol recommended by the manufacturer was followed for the analysis. A standard curve with seven points was generated for each cytokine. Serum matrix was added to the standard, control, and blank wells to mimic the SP composition. Two controls were added in their corresponding wells. The microsphere solution was sonicated, vortexed, and added to each well of controls, standards, and SP samples. The plates, in continuous stirring, were kept in the dark at 4 °C for 16–18 h. Thereafter, the contents of each well were removed, and they were washed thrice with the washing solution. Then, detection antibodies were added to the wells and the plates were kept at RT in the dark for 2 h. Thereafter, streptavidin–phycoerythrin was added to the wells and the plates were incubated at RT for 30 min. Then, the wells were washed, and the plates were run in a MAGPIX R (Luminexcorp, Austin, TX, USA). Data were acquired with xPONENT software version 4.2 (Luminexcorp) and analyzed using MILLIPLEX R Analyst Version 5.1 (Merck Millipore). The SP concentration of each cytokine, expressed as pg/mL, was obtained by measuring the average fluorescent intensity in a 5-parameter logistic curve-fitting.

### 2.3. Experimental Design

The experiment evaluated the variability among breeding boars (inter-boar variability) and within the same boar (intra-boar variability) in the concentrations of cytokines measured in SP samples throughout the year. The study also tested the hypothesis that variations in ambient daylight and temperature in the region where the animals were held could explain the intra-boar variability of cytokine levels in SP. Therefore, a total of 180 ejaculates were collected over one year from nine boars, at the rate of 20 ejaculates per boar with at least one ejaculate collected per boar per month. The SP samples were grouped according to the two dominating daylength and accompanying temperature periods of the year, characteristic of the geographical place where the animals were held. Namely, increasing, from January to June, and decreasing, from July to December. A total of 90 SP samples, 10 per boar, were analyzed per each ambient daylength/temperature period.

### 2.4. Statistical Analysis

The IBM SPSS Statistics package (IBM Spain, Madrid) version 24.0 was used for statistical analysis. The Shapiro–Wilks test of standardized residual data was done for checking the assumption of normality, and data not normally distributed were square root transformed. First, a one-way ANOVA was performed to assess the variability between boars (1 to 9). Then, the intraclass correlation coefficient (ICC), using a two-way mixed-effect model (3,1), was used for measuring the relevance of intra-boar variance relative to the total [26]. ICC values range from 0 to 1, and values less than 0.5 are considered indicative of poor reliability, between 0.5 and 0.75 of moderate reliability, between 0.75 and 0.9 of good reliability, and greater than 0.90 of excellent reliability [27]. Once intra-boar reliability was shown to be high, a two-way multivariate analysis of variance (two-way MANOVA) was performed to determine the effects of boar (1 to 9) and year period of ejaculate collection (increasing vs. decreasing periods) on SP-cytokine concentrations and ejaculate parameters. Thereafter, since the interaction of boar per season was significant, the effect of year period on SP-cytokine concentrations on each boar was evaluated using one-way ANOVA. The Tukey test was used for post-hoc analyses, and statistical significance was considered from *p* < 0.05. Data were showed as the means ± standard error of the mean (SEM). The Spearman rank correlation coefficient was used to evaluate possible relationships between SP cytokines (concentration and total amount) and the ejaculate parameters (volume, sperm concentration and total number of spermatozoa). Only the relationships that explained a substantial proportion of the variance in the SP cytokines were considered, specifically, those with a correlation coefficient (R value) greater than 0.70, which is indicative of explaining more than 50% of the variance [28].

## 3. Results

**Figure 1:**
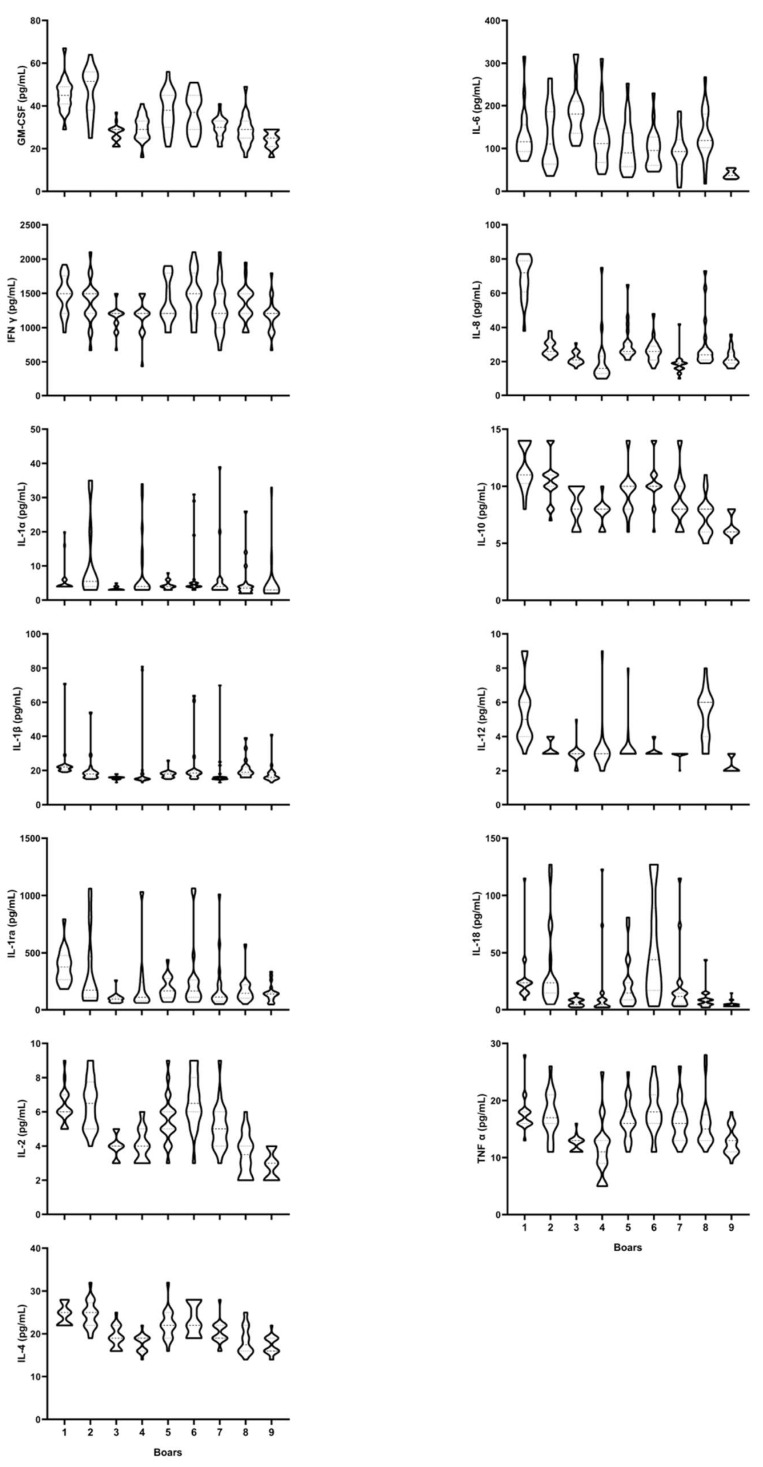
Violin plots representing the concentrations of granulocyte macrophage colony-stimulating factor (GM-CSF), interferon-gamma (IFNγ), interleukin (IL)-1α, IL-1β, IL-1ra, IL-2, IL-4, IL-6, IL-8, IL-10, IL-12, IL-18, and tumor necrosis factor-α (TNFα) in seminal plasma of nine boars (20 ejaculates per boar). The dashed line represents the median and the dotted lines the 25% and 75% quartiles. All cytokines showed differences among boars (*p* < 0.001).

**Figure 2:**
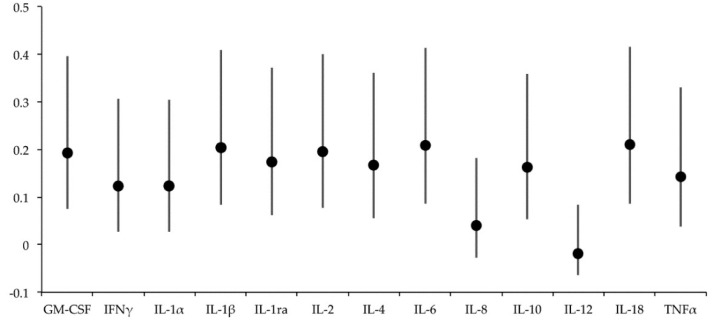
Intraclass correlation coefficient (ICC 3,1) values in terms of single measures (dot) and 95% confidence intervals (bars) of cytokine concentrations in 180 boar seminal plasma samples. Cytokines: granulocyte-macrophage colony-stimulating factor (GM-CSF), interferon-gamma (IFNγ), interleukin (IL)-1α, IL-1β, IL-1ra, IL-2, IL-4, IL-6, IL-8, IL-10, IL-12, IL-18, and tumor necrosis factor-α (TNFα).

**Figure 3:**
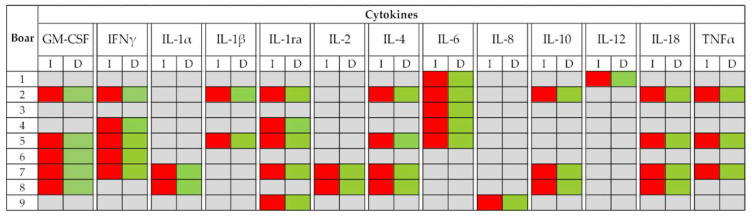
Variation pattern of cytokine concentrations in seminal plasma from ejaculates collected from nine boars (20 ejaculates per boar) during the times of year when increasing (I, January to June) or decreasing daylight/temperature (D, July to December) dominated. Grey box indicates no differences between periods, while red and green boxes indicate a relationship between dominating seasonal parameters with high and low cytokine concentrations (*p* < 0.05), respectively. Cytokines: Granulocyte macrophage colony-stimulating factor (GM-CSF), interferon-gamma (IFNγ), interleukin (IL)-1α, IL-1β, IL-1ra, IL-2, IL-4, IL-6, IL-8, IL-10, IL-12, IL-18, and tumor necrosis factor-α (TNFα).

All measured cytokines showed differences (*p* < 0.001) in SP concentrations among boars (Figure 1). The ICC (3,1) values were low for all the measured cytokines, as they ranged from −0.02 to 0.21 (Figure 2), which was indicative of poor reliability and larger within-boar than between-boar variability. In addition to the significant effect of boar on SP-cytokine concentrations (V = 0.005, F(104, 1037) = 11.674, *p* < 0.001), Wilks’ Lambda test revealed a significant effect of the chosen increasing or decreasing period for ejaculate collection (V = 0.458, F(13, 149) = 13.548, *p* = 0.001) and also a significant interaction between boar and period (V = 0.210, F(104, 1037) = 2.537, *p* = 0.001). The nine boars experienced differences between the two daylength/temperature periods for at least one SP cytokine. The boars numbered 2, 5, and 7 showed differences in 8 or more SP cytokines, while those numbered as 1, 3, 6, and 9 showed differences only in two or less SP cytokines. The pattern of variation between the ambient daylength/temperature periods was similar, irrespective of boar or cytokine, and it was characterized by higher (*p* < 0.05) SP-cytokine concentrations in the increasing than in the decreasing period (Figure 3). The SP cytokines most influenced by daylength period were GM-CSF, IFNγ, IL-1ra, and IL-6, as their SP concentrations differed between the two daylength/temperature periods in five boars. The data of the SP-cytokine concentrations within each daylength/temperature period for each one of the nine boars appear in Table S1.

**Figure 4:**
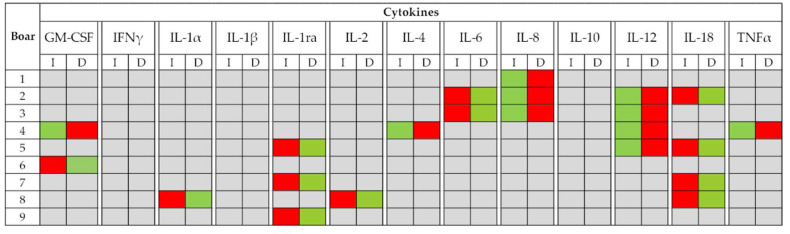
Variation pattern in the total amount of seminal plasma cytokines in ejaculates collected from nine boars (20 ejaculates per boar) during the periods of increasing (I, January to June) or decreasing daylight/temperature (D, July to December). Grey box indicates no differences between periods, while red and green boxes indicate a relationship between dominating seasonal parameters with high and low total cytokine amounts (*p* < 0.05), respectively. Cytokines: Granulocyte macrophage colony-stimulating factor (GM-CSF), interferon-gamma (IFNγ), interleukin (IL)-1α, IL-1β, IL-1ra, IL-2, IL-4, IL-6, IL-8, IL-10, IL-12, IL-18, and tumor necrosis factor-α (TNFα).

The volume of each ejaculate was registered to express the total amount of cytokines per ejaculate. Ejaculate volume was larger (*p* < 0.01) during decreasing than increasing daylength/temperature periods in all nine boars (Table 1), with the total SP amount of some cytokines, specifically between boars 1 and 4, also differing between periods in all nine boars. The total SP amount of three cytokines, specifically IFNγ, IL-1β, and IL-10, did not show significant differences between periods in any boar. The pattern of variation displayed by the total amounts of cytokines in SP varied between periods (Figure 4), all data are displayed per boar in Table S2.

In addition to ejaculate volume, sperm concentration and the total number of spermatozoa per ejaculate were also explored for possible relationships with SP-cytokine levels. Similar to ejaculate volume, both sperm concentration and total sperm number were also higher in the decreasing daylength/temperature period (Table 1). Only ejaculate volume was strongly related (R > 0.7) to total cytokine amount for some cytokines, an expected relationship considered of trivial value since ejaculate volume is the variable that determines total cytokine amount per ejaculate. The other relationships between SP cytokines and ejaculate parameters barely explained between 0% and 20% of total variance (Table S3).

## 4. Discussion

The present study confirmed the hypothesis tested: the period of the year of ejaculate collection influenced the concentration and the total amount of SP cytokines. This is one of the causes that helps explain the great intra-male variability in SP-cytokine levels.

The first result of the present study verified the variability between males (inter-male) in the SP concentration of all measured cytokines, already known in pigs [19] and in males of other mammalian species such as humans [18]. Such inter-male variability opens up the possibility of using the SP concentration of some cytokines as a diagnostic tool for some male reproductive disorders [29,30,31], ejaculate parameters [16,32], and even to predict the ability of spermatozoa to withstand some biotechnological procedures, such as cryopreservation [33]. However, the results of the present study, demonstrating a substantially higher intra-male variability than inter-male variability, weaken the practical usefulness of SP-cytokine concentrations as a diagnostic tool. The existence of intra-male differences in other bioactive SP components has been previously documented in pigs, being particularly noticeable for total and specific protein concentrations [34] and for components with antioxidant properties, such as glutathione peroxidase [35] or melatonin [36]. Similarly, fluctuations in SP-cytokine concentrations over time were reported in healthy men [18,37]. The causes for these fluctuations have not been explained.

In this context, this study explored whether the period of the year in which ejaculates were collected would be one of the causal factors that explain the substantial variability among male pigs in SP-cytokine levels. The study was conducted in European pigs, a species of mammal that is generally not considered reproductively seasonal, but still shows a tangible seasonal reproductive pattern [38]. This seasonal pattern has been well documented in breeding boars, influencing sperm production and quality [39,40,41,42,43]. The four seasons of the year in temperate geographic areas, namely winter, spring, summer, and autumn, are environmentally mainly characterized by differences in daylight and in air temperature. Fluctuations in daylight are considered the main environmental factor influencing the reproductive performance in pigs [41,44,45]. In fact, daylight fluctuation is one of the main causes for discarding ejaculates in AI boars, finding the highest proportions of unusable ejaculates, due to poor sperm quality, during the annual period with decreasing daylength [46,47]. However, the ambient temperature is an additional factor, since it basically follows ambient daylight and is a major problem when dealing with the comfort of the pigs under production. The hot air temperature usual during summer in the Mediterranean area, where the boars were housed, causes heat stress in livestock, with severe lasting negative effects on sperm quality affecting ejaculates collected during summer and autumn, even in breeding boars housed under controlled environmental conditions [22,23,24]. The relevance of environmental temperature in the quantity and seminal quality of boars housed under controlled conditions varies according to geographic areas. Therefore, it can be practically insignificant in areas with limited variations in air temperature [48] and relevant in those areas with large variations, particularly in those areas that have periods of high air temperatures [25], such as in the Mediterranean area during summer. Therefore, environmental management has been developed and today must follow European laws in order to preserve their health and welfare, including artificial lighting and evaporative air-measures. Despite the breeding boars included in this study being confined under these prerogatives, our results confirm the existence of a seasonal influence on SP-cytokine levels, accompanying other semen variables already described and listed above. The worst sperm quality was depicted by the ejaculates collected during the decreasing daylength/temperature period, as evidenced by the increased percentage of sperm morphological abnormalities. Thus, the classical four seasons of the year were grouped into two annual periods according to fluctuations in ambient daylight and temperature, one with increasing daylight/temperature (winter and spring) and another with decreasing daylight/temperature (summer and autumn).

By grouping the SP samples into these two annual periods, the present study demonstrated, for the first time in any livestock species, that the period of the year when ejaculates were collected influenced the SP levels of cytokines. The seminal concentration of all cytokines differed between the two periods of the year following the same pattern of variation in all breeding boars. The highest concentrations were measured in the increasing daylength/temperature period. The SP cytokines GM-CSF, IFNγ, IL-1ra, and IL-6 were the most influenced by the period of ejaculate collection because their concentrations differed between periods in most breeding boars. In contrast, IL-1α, IL-1β, IL-2, IL-8, and IL-12 were the least influenced. In addition to concentration, differences in the total amount of some SP cytokines were also observed between the daylength/temperature periods. This clear difference between male pigs in the sensitivity of SP cytokines to environmental conditions was consistent with previous studies in men showing that the range of intra-male variation in SP cytokines over time, differs between cytokines [18,37]. Similarly, the number of SP cytokines showing differences between daylength/temperature periods varied among boars, as it ranged from one to nine. This finding should be considered reasonable from the existing knowledge of individual variability in the tolerance of boars to annual fluctuations in environmental conditions, with some being more sensitive than others, which impacts reproductive seasonality [38,49,50]. Needless to say, this individual variability was not related to disease or age of the boars, since all the boars were healthy and were of similar age throughout; perhaps it could be related to individual heat stress. Rempel et al. [42] highlighted that boars with high genetic merit would have a higher thermal sensitivity and could experience heat stress due to high air temperatures in summer, even when housed in AI centers under controlled environmental conditions.

To the best of our knowledge, there are no studies that identify which factors mediate the effect of environmental conditions on SP-cytokine levels and, consequently, which could explain why the SP levels of many cytokines differed between the annual periods of ejaculate collection. Similar to the fluctuation we found with cytokines, other authors have also found annual fluctuations for other bioactive SP components in breeding boars used in AI programs, for example, in melatonin [36], proteins [51], or enzymes with antioxidant properties [52]; yet none of them provided sound explanations about the physiological mechanisms behind such annual fluctuations. Therefore, the matter remains open to assumptions and further discussion. Cytokines are widely present in all reproductive organs of male pigs, including testes, epididymides, and accessory sex glands [53,54,55], and they are also synthetized by non-immune cells [30]. Therefore, the reproductive tissues have the ability to release the synthetized cytokines to the lumen of the genital tract, including the SP formed at ejaculation. Therefore, the annual fluctuation in functionality undergone by the boar reproductive organs should explain the annual fluctuation in SP levels of cytokines. Hormones, particularly steroids, and within these mainly androgens, regulate the functionality of the male reproductive organs, including spermatogenesis, and annual fluctuations in environmental conditions are one of the main factors influencing corresponding changes in both blood and seminal androgens levels [45]. The fluctuation in androgens closely and positively relates to annual fluctuations in ejaculate volume and sperm concentration [56]. The lack of a biologically sound relationship between semen parameters and SP-cytokine levels would indicate that cellular mechanisms and/or signaling molecules regulating SP cytokines under physiological conditions may be independent from that regulating semen production. Thus, other drivers beyond steroids (mainly androgens) may be responsible for mediating the environmental effect on the SP levels of cytokines. The annual fluctuations in environment conditions also affect levels of blood circulating cytokines in healthy men, sparking controversy about which molecular mediators are the effective ones [21]. In this regard, the driving mediators between environmental conditions and circulating blood cytokines do not have to be the same for SP cytokines because the cytokine levels of blood and SP are unrelated, at least in men [57]. Consequently, further research is needed to elucidate how annual fluctuations in environmental conditions influence the SP levels of cytokines.

## 5. Conclusions

In summary, the present study demonstrated that the variation in SP levels of cytokines within individual breeding boars is greater than that between breeding boars and that annual fluctuations in daylength and air temperature contribute in explaining this intra-male variability. Further studies are required to investigate the clinical and biological relevance of annual fluctuations for the reproductive health of male pigs and also to elucidate the molecular mechanisms that drive the environmental effects.

## Figures and Tables

**Table 1:** Semen variables (mean ± SEM) of the 180 ejaculates used in the experiment (90 ejaculates per daylength/temperature period).

Ejaculate Variables	Overall	Daylength/Temperature Period of the Year ^1^		*p* Value ^2^
		Increasing	Decreasing	
Volume (mL)	212.41 ± 4.98	190.33 ± 5.05	234.99 ± 7.98	<0.001
Sperm concentration (×10^6^ sperm/mL)	259.92 ± 2.15	227.86 ± 3.09	294.10 ± 3.40	<0.001
Total sperm number (×10^9^ spermatozoa)	56.35 ± 1.96	43.77 ± 1.71	68.27 ± 2.62	<0.001
Spermatozoa with normal morphology (%)	87.35 ± 0.93	92.80 ± 1.88	82.11 ± 2.19	0.017

^1^ Increasing daylength/temperature: from January to June; decreasing daylength/temperature: from July to December. ^2^ Significance of differences between increasing and decreasing daylength/temperature.

## Supplementary Materials

The following are available online at https://www.mdpi.com/2079-7737/9/5/105/s1, Table S1: Concentration of cytokines (pg/mL; mean ± SEM) in seminal plasma of ejaculates (n: 180) collected in nine boars (20 ejaculates per boar) during periods of increasing (I, January to June) and decreasing ambient daylength and air temperature (D, July to December). Table S2: Total amount of cytokines (ng/mL; mean ± SEM) in seminal plasma of ejaculates (n: 180) collected in nine boars (20 ejaculates per boar) during the periods of increasing (I, January to June) and decreasing ambient daylength and air temperature (D, July to December). Table S3: Relationship between seminal plasma cytokines and ejaculate parameters.
