# Supplementary material for: Period of Boar Ejaculate Collection Contributes to the Yearly Intra-Male Variability of Seminal Plasma Cytokines
Source: Biology (Basel). 2020 May 20;9(5):105. doi: 10.3390/biology9050105 (PMC7285058; doi:10.3390/biology9050105)
Supplement: Supplementary file 1 [file biology-09-00105-s001.pdf]

Supplementary Table S1. Concentration of cytokines (pg/mL; mean±SEM) in seminal plasma of ejaculates (n: 180) collected in nine boars (20 ejaculates per boar) during the year periods of increasing daylength (ID, January to June) and decreasing daylength (DD, July to December).

Cytokines[1]	Daylight period	Boar								
		1	2	3	4	5	6	7	8	9
GM-CSF	ID	48.00±2.55	55.60±1.31[a]	27.80±1.04	28.10±2.03	42.70±2.72[a]	42.80±1.99[a]	33.20±1.28[a]	32.60±2.10[a]	24.50±1.46
	DD	42.20±2.24	38.50±2.75[b]	26.20±1.58	31.80±1.58	33.40±2.56[b]	30.20±2.39[b]	27.00±1.23[b]	24.50±1.78[b]	23.90±1.23
IFNγ	ID	1540.30±74.92	1587.40±77.80[a]	1113.10±71.07	1322.60±78.08[a]	1609.90±94.66[a]	1652.00±64.76[a]	1500.70±98.81[a]	1457.20±92.41	1185.90±98.01
	DD	1413.40±94.44	1156.00±80.32[b]	1180.70±46.05	1019.40±47.19[b]	1153.10±56.08[b]	1344.20±119.94[b]	1100.80±84.74[b]	1238.50±66.08	1181.00±54.69
IL-1α	ID	5.60±1.19	11.40±3.07	3.60±0.27	11.40±3.91	4.80±0.47	6.00±1.46	9.40±3.65[a]	7.10±2.34[a]	4.00±0.93
	DD	6.00±1.58	10.40±4.03	3.20±0.13	5.30±1.23	3.80±0.29	9.20±3.48	3.60±0.31[b]	3.30±0.78[b]	5.80±3.03
IL-1β	ID	27.00±4.96	23.40±3.58[a]	15.90±0.28	28.30±8.62	19.10±0.82[a]	23.10±4.56	22.90±5.35	21.70±2.11	19.20±2.52
	DD	21.30±0.45	16.90±0.48[b]	15.60±0.40	15.80±0.61	16.60±0.34[b]	22.60±4.43	15.20±0.29	20.20±1.62	16.80±0.77
IL-1ra	ID	439.10±48.73	513.30±115.25[a]	117.90±17.51	335.10±117.65[a]	253.50±30.06[a]	217.10±37.00	306.20±90.40[a]	191.80±47.80	190.50±27.13[a]
	DD	325.40±38.45	155.50±37.43[b]	87.30±6.44	125.70±30.72[b]	120.30±12.27[b]	323.60±121.43	87.40±9.25[b]	149.60±14.82	107.40±12.11[b]
IL-2	ID	6.60±0.37	7.40±0.40	4.00±0.26	4.00±0.33	6.10±0.53	6.70±0.34	6.00±0.39[a]	4.30±0.30[a]	3.00±0.26
	DD	6.00±0.26	5.50±0.31	3.90±0.18	4.10±0.31	5.10±0.31	6.60±0.64	4.30±0.26[b]	2.60±0.27[b]	2.70±0.26
IL-4	ID	24.60±0.79	26.60±0.87[a]	19.30±1.04	17.60±0.75	23.60±1.28[a]	24.10±1.01	21.70±0.83[a]	20.50±1.03[a]	17.60±0.75
	DD	24.40±0.75	22.40±0.65[b]	19.00±0.63	18.20±0.49	20.80±0.66[b]	22.30±1.30	19.00±0.63[b]	16.70±0.70[b]	17.00±0.58
IL-6	ID	168.60±19.88[a]	188.70±14.83[a]	219.70±19.93[a]	157.30±22.90[a]	142.60±18.24[a]	112.50±17.68	85.90±16.94	137.10±24.05	40.50±3.05
	DD	95.50±5.22[b]	67.70±5.52[b]	150.40±11.37[b]	88.30±10.17[b]	67.30±8.84[b]	86.00±10.09	101.70±11.85	125.30±11.03	39.20±3.00
IL-8	ID	65.40±4.45	29.90±1.64	20.20±0.81	24.30±6.33	29.50±2.36	28.60±2.40	20.40±2.63	23.50±1.07	24.70±1.90[a]
	DD	73.40±2.86	26.20±1.03	23.20±1.28	16.00±1.51	31.70±4.12	24.10±1.88	16.90±0.78	35.00±6.00	19.50±0.90[b]
IL-10	ID	11.80±0.66	11.70±0.52[a]	8.20±0.55	7.80±0.36	10.20±0.70	10.30±0.54	10.30±0.70[a]	8.30±0.52[a]	6.50±0.34
	DD	11.50±0.58	9.30±0.45[b]	8.60±0.52	7.60±0.40	8.70±0.47	10.50±0.72	7.40±0.43[b]	6.40±0.37[b]	6.60±0.31
IL-12	ID	6.30±0.67[a]	3.50±0.17	2.70±0.15	3.30±0.65	3.50±0.50	3.10±0.10	2.90±0.10	5.00±0.45	2.10±0.10
	DD	4.50±0.27[b]	3.10±0.10	3.20±0.20	3.10±0.10	3.10±0.10	3.20±0.13	3.00±0.00	5.50±0.48	2.50±0.17
IL-18	ID	34.40±9.57	54.70±13.04[a]	7.20±1.17	23.90±13.02	34.80±8.16[a]	56.50±11.54	31.50±11.05[a]	13.90±3.51[a]	5.70±1.17
	DD	19.80±1.80	14.00±2.00[b]	6.30±1.30	6.70±1.33	9.70±1.90[b]	50.40±16.87	7.40±1.42[b]	4.40±0.65[b]	4.40±0.60
TNFα	ID	18.30±1.33	20.30±0.92[a]	12.30±0.52	9.20±1.01	18.60±1.14[a]	18.20±1.30	18.80±0.99[a]	16.50±1.42	13.10±0.89
	DD	17.00±0.33	14.60±0.76[b]	12.50±0.34	13.60±1.78	14.60±0.60[b]	18.30±1.33	13.70±0.54[b]	15.20±1.37	12.60±0.64

Cytokines: Growth factor granulocyte–macrophage colony-stimulating factor (GM-CSF), interferon (IFN), interleukin (IL) and tumor necrosis factor (TNF). a,b: indicates differences to $p < 0.05$ between D and DD in ach boar.

Supplementary Table S2. Total amount of cytokines (ng/mL; mean±SEM) in seminal plasma of ejaculates (n: 180) collected in nine boars (20 ejaculates per boar) during the year periods of increasing daylength (ID, January to June) and decreasing daylength (DD, July to December).

Cytokines[1]	Daylight period	Boar								
		1	2	3	4	5	6	7	8	9
GM-CSF	ID	12.14±1.52	12.80±1.12	5.14±0.27	4.60±0.46[b]	7.69±0.53	7.77±0.48[a]	5.28±0.39	5.94±0.60	4.61±0.25
	DD	13.17±1.17	13.89±1.35	5.18±0.37	7.66±1.24[a]	7.63±0.52	6.48±0.79[b]	5.35±0.44	4.64±0.47	4.45±0.37
IFNγ	ID	383.15±41.16	364.86±34.66	203.11±11.58	218.36±15.10	285.71±23.01	298.87±15.25	236.19±17.06	264.66±24.25	219.77±10.88
	DD	455.12±43.23	414.13±36.01	232.31±11.24	232.54±38.74	267.99±18.34	285.06±31.32	220.88±26.72	228.95±15.38	218.68±16.95
IL-1α	ID	1.46±0.41	2.72±0.83	0.66±0.05	2.04±0.73	0.86±0.09	1.10±0.28	1.46±0.53	1.27±0.42[a]	0.73±0.15
	DD	1.80±0.36	3.47±1.36	0.63±0.03	1.14±0.25	0.88±0.07	2.15±0.91	0.71±0.06	0.61±0.14[b]	1.23±0.73
IL-1β	ID	7.04±1.79	5.56±1.15	2.92±0.08	4.84±1.61	3.32±0.16	4.22±0.89	3.58±0.75	3.87±0.38	3.59±0.40
	DD	6.74±0.52	5.98±0.27	3.07±0.13	3.61±0.41	3.87±0.25	5.12±1.43	2.99±0.18	3.76±0.35	3.17±0.26
IL-1ra	ID	107.34±13.18	117.56±28.07	21.44±3.02	59.27±21.70	41.97±5.15[a]	38.71±5.88	47.10±13.05[a]	33.72±8.47	35.99±5.13[a]
	DD	112.70±18.68	56.07±15.76	17.35±1.56	27.38±6.54	28.69±4.15[b]	75.26±31.92	17.09±2.00[b]	27.95±3.22	20.14±2.59[b]
IL-2	ID	1.65±0.19	1.70±0.16	0.74±0.05	0.66±0.07	1.10±0.12	1.21±0.06	0.94±0.07	0.79±0.08[a]	0.56±0.04
	DD	1.92±0.13	1.98±0.17	0.77±0.04	1.00±0.17	1.17±0.07	1.41±0.17	0.86±0.10	0.49±0.06[b]	0.51±0.07
IL-4	ID	6.13±0.63	6.10±0.50	3.55±0.22	2.89±0.23[b]	4.19±0.24	4.36±0.25	3.42±0.19	3.72±0.29	3.32±0.12
	DD	7.72±0.62	8.04±0.52	3.74±0.17	4.24±0.55[a]	4.85±0.32	4.76±0.44	3.79±0.32	3.12±0.22	3.20±0.24
IL-6	ID	42.87±7.37	41.90±2.49[a]	40.27±3.77[a]	27.07±5.27	21.69±1.96	20.79±3.80	14.72±3.46	24.30±4.19	7.75±0.72
	DD	30.80±3.51	23.57±1.52[b]	29.73±2.51[b]	19.48±3.45	15.63±2.03	17.91±2.00	20.02±2.20	23.60±2.49	7.52±0.76
IL-8	ID	16.58±2.13[b]	6.82±0.57[b]	3.74±0.24[b]	4.19±1.29	5.11±0.39	5.09±0.30	3.22±0.41	4.23±0.26	4.76±0.47
	DD	23.48±2.29[a]	9.37±0.65[a]	4.59±0.32[a]	3.92±0.65	7.73±1.54	5.18±0.64	3.39±0.31	6.35±1.03	3.68±0.30
IL-10	ID	2.98±0.36	2.74±0.32	1.50±0.10	1.31±0.14	1.79±0.15	1.87±0.12	1.64±0.14	1.51±0.14	1.22±0.05
	DD	3.72±0.40	3.33±0.24	1.69±0.11	1.79±0.24	2.01±0.12	2.24±0.23	1.48±0.16	1.19±0.08	1.22±0.09
IL-12	ID	1.57±0.22	0.81±0.09[b]	0.50±0.03[b]	0.56±0.14[b]	0.60±0.08[b]	0.56±0.03	0.47±0.04	0.90±0.10	0.40±0.02
	DD	1.40±0.13	1.11±0.07[a]	0.63±0.05[a]	0.70±0.08[a]	0.72±0.05[a]	0.68±0.06	0.59±0.03	1.06±0.13	0.47±0.05
IL-18	ID	9.13±3.17	12.49±3.17[a]	1.32±0.22	4.282.50	6.63±1.50[a]	9.77±1.81	4.93±1.64[a]	2.52±0.64[a]	1.03±0.18
	DD	6.52±0.78	4.99±0.80[b]	1.26±0.27	1.71±0.41	2.17±0.37[b]	11.12±4.01	1.52±0.36[b]	0.82±0.13[b]	0.84±0.16
TNFα	ID	4.59±0.60	4.68±0.44	2.26±0.11	1.50±0.19[b]	3.30±0.18	3.28±0.26	3.01±0.26	3.03±0.38	2.44±0.10
	DD	5.39±0.45	5.27±0.43	2.450.08	3.45±0.81[a]	3.36±0.14	3.92±0.45	2.73±0.24	2.78±0.20	2.38±0.20

Cytokines: Growth factor granulocyte–macrophage colony-stimulating factor (GM-CSF), interferon (IFN), interleukin (IL) and tumor necrosis factor (TNF). a,b: indicates differences to $p < 0.05$ between ID and DD in ach boar.

Supplementary Table S3. Relationship between seminal plasma (SP) cytokines and ejaculation parameters. The results are shown as correlation coefficient and percentage of explained variance (in brackets). In grey, the relationships explaining more than 50% of the total variance.

Cytokine	SP Cytokine concentration			Total SP amount of cytokines		
	Ejaculate volume	Sperm concentration	Total sperm count	Ejaculate volume	Sperm concentration	Total sperm count
GM-CSF	0.224** (5.02)	-0.275** (7.56)	-0.081 (0.66)	0.674** (45.42)	-0.159* (2.53)	0.251** (6.30)
IFNγ	-0.059 (0.35)	-0.204** (4.16)	-0.193* (3.72)	0.616** (37.9)	-0.115 (1.32)	0.257** (6.60)
IL-1α	0.043 (0.18)	-0.272** (7.40)	-0.198** (3.92)	0.518** (26.83)	-0.195** (3.80)	0.128 (1.64)
IL-1β	0.061 (0.37)	-0.231** (5.33)	-0.141 (2.00)	0.685** (46.92)	-0.111 (1.23)	0.287** (8.24)
IL-1ra	-0.004 (0.0016)	-0.294** (8.64)	-0.235** (5.52)	0.323** (10.43)	-0.269** (7.23)	-0.030 (0.09)
IL-2	0.215** (4.62)	-0.312** (9.73)	-0.141 (1.99)	0.624** (38.94)	-0.221** (4.88)	0.169* (2.86)
IL-4	0.212** (4.50)	-0.296** (8.76)	-0.111 (1.23)	0.769** (59.14)	-0.125 (1.56)	0.317** (10.05)
IL-6	-0.066 (0.44)	-0.119 (1.42)	-0.136 (1.85)	0.338** (11.42)	-0.111 (1.23)	0.104 (1.08)
IL-8	0.333** (11.08)	-0.185* (3.42)	0.025 (0.06)	0.670** (44.89)	-0.107 (1.14)	0.265** (7.02)
IL-10	0.223** (4.97)	-0.261** (6.81)	-0.086 (0.74)	0.694** (48.16)	-0.144 (2.07)	0.268** (7.18)
IL-12	0.223** (4.97)	0.023 (0.05)	0.164* (2.69)	0.718** (51.55)	0.060 (0.36)	0.444** (19.71)
IL-18	0.109 (1.19)	-0.358** (12.82)	-0.230** (5.29)	0.320** (10.24)	-0.322** (10.37)	-0.082 0.67
TNFα	0.088 (0.77)	-0.177* (3.13)	-0.103 (1.06)	0.664** (44.09)	-0.099 (0.98)	0.288** (8.29)

*$p < 0.05$; **$p < 0.01$
